# Lipid regulation of the glucagon receptor family

**DOI:** 10.1530/JOE-23-0335

**Published:** 2024-05-06

**Authors:** Affiong Ika Oqua, Yusman Manchanda, Emma Rose McGlone, Ben Jones, Sarah Rouse, Alejandra Tomas

**Affiliations:** 1 Section of Cell Biology and Functional Genomics, Division of Diabetes, Endocrinology and Metabolism, Department of Metabolism, Digestion and Reproduction, Imperial College London, London, UK; 2 Department of Surgery and Cancer, Imperial College London, London, UK; 3 Section of Investigative Medicine, Division of Diabetes, Endocrinology and Metabolism, Department of Metabolism, Digestion and Reproduction, Imperial College London, London, UK; 4 Department of Life Sciences, Imperial College, London, UK

**Keywords:** lipid, glucagon receptor, GLP-1 receptor, GIP receptor, cholesterol

## Abstract

The glucagon receptor family are typical class B1 G protein-coupled receptors (GPCRs) with important roles in metabolism, including the control of pancreas, brain, and liver function. As proteins with seven transmembrane domains, GPCRs are intimately in contact with lipid bilayers and therefore can be putatively regulated by interactions with their lipidic components, including cholesterol, sphingolipids, and other lipid species. Additionally, these receptors, as well as the agonists they bind to, can undergo lipid modifications, which can influence their binding capacity and/or elicit modified or biased signalling profiles. While the effect of lipids, and in particular cholesterol, has been widely studied for other GPCR classes, information about their role in regulating the glucagon receptor family is only beginning to emerge. Here we summarise our current knowledge on the effects of cholesterol modulation of glucagon receptor family signalling and trafficking profiles, as well as existing evidence for specific lipid–receptor binding and indirect effects of lipids via lipid modification of cognate agonists. Finally, we discuss the different methodologies that can be employed to study lipid–receptor interactions and summarise the importance of this area of investigation to increase our understanding of the biology of this family of metabolically relevant receptors.

## Introduction

Lipids are structurally diverse organic compounds crucial for the normal functioning of all cells, as they are key constituents of membranes essential for cellular structure that enable the compartmentalisation of tightly regulated processes both within the cell and in intracellular organelles. Lipids are also major contributors to intracellular signalling, either via lipidic post-translational modifications or as allosteric modulators of membrane receptors and other factors, as well as being important sources of energy, stored within cells as lipid droplets (Shevchenko & Simons 2010, Klose *et al*. 2013, Muro *et al*. 2014, Buenaventura *et al*. 2019, Damian *et al*. 2021). The structural characteristics of the lipids present in membranes determines their mechanical properties, controlling parameters ranging from membrane fluidity and curvature to thickness and shape, as well as the propensity for the formation of rafts and other signalling nanodomains. Unique combinations of membrane lipids and proteins allow for the development of cell/organelle-specific functions, ranging from those of the plasma membrane and/or trafficking vesicles to mitochondrial membranes harbouring electron transport chains (Guo *et al*. 2018), or the protein folding and transport functions of endoplasmic reticulum (ER) membranes (Phillips & Miller 2021), amongst others (Shevchenko & Simons 2010, Klose *et al*. 2013, Muro *et al*. 2014, Bolla *et al*. 2019).

Incretins, characterised by Creutzfeldt in 1979, are peptide hormones secreted in the gastrointestinal tract in response to nutrients that potentiate the glucose-dependent secretion of insulin. The primary incretins include glucagon-like peptide 1 (GLP-1), secreted mainly from enteroendocrine L cells, with its truncated versions, GLP-1(7–37) and GLP-1(7–36)_NH2_, binding to and activating cognate GLP-1 receptor (GLP-1R) (Donnelly, 2012, Campbell & Drucker 2013, Paternoster & Falasca 2018), and glucose-dependent insulinotropic polypeptide (GIP), a 42-amino acid peptide (GIP(1–42)) secreted from enteroendocrine K cells that binds to and activates the GIP receptor (GIPR) (Creutzfeldt 1979, Campbell & Drucker 2013, Chia & Egan 2020). Glucagon is a closely related peptide which derives, like GLP-1, from pre-proglucagon (Müller *et al*. 2017) and binds to the glucagon receptor (GCGR). It is released from pancreatic alpha cells and it is best known for its ability to increase hepatic glucose production at times of hypoglycaemia (Marroqui *et al*. 2014). Although glucagon is not considered an incretin, it has some similar features as it is secreted in response to protein ingestion (Müller *et al*. 1970, Day *et al*. 1978, Ang *et al*. 2019) and is insulinotropic (Capozzi *et al*. 2019*a*, *b*). Although not usually associated with type 2 diabetes or obesity, the glucagon family of receptors also includes the GLP-2 receptor (GLP-2R), which is activated by GLP-2, a 33-amino acid peptide (GLP-2(1–33)) also derived from pre-proglucagon and secreted mainly from enteroendocrine L cells, like GLP-1. GLP-2 promotes intestinal growth and enhances nutrient absorption amongst other functions (Bahrami *et al*. 2010, Drucker & Yusta 2014, Gadgaard *et al*. 2023).

The GLP-1R is found mainly in pancreatic beta cells and hypothalamic neurons. Besides its endogenous activation by GLP-1, pharmacological targeting of the GLP-1R is achieved with a range of peptide agonists, including exendin-4 and semaglutide, amongst others (Campbell & Drucker 2013, Knudsen & Lau 2019). The main function of the GLP-1R is the regulation of blood glucose levels through the potentiation of insulin secretion in a glucose-dependent manner and the control of appetite through the regulation of neuronal feeding centres (Campbell & Drucker 2013, Boer & Holst 2020). The best characterised GLP-1R intracellular signalling cascade involves coupling of the active receptor to Gαs, resulting in the activation of adenylate cyclase and leading to the generation of cyclic adenosine monophosphate (cAMP). This in turn leads to activation of protein kinase A (PKA) and exchange protein activated by cAMP 2 (Epac2), followed by engagement of diverse downstream signalling networks, culminating in an increase in insulin synthesis and secretion as well as promotion of beta cell survival (Holst *et al*. 2009, Boer & Holst 2020, El Eid *et al*. 2022).

Expression of GIPR partly overlaps with that of GLP-1R, for example, in pancreatic islets and some anorectic brain regions (albeit in mainly distinct neuronal subpopulations), but GIPR is also found in non-overlapping tissues such as bone and adipose tissue although there is some uncertainty on the exact cell type that expresses the GIPR in the latter (Yip *et al*. 1998, Kim *et al*. 2013, Campbell *et al*. 2022). Besides activation by its cognate hormone GIP, the GIPR is activated by pharmacological peptide agonists such as the GLP-1R/GIPR dual agonist tirzepatide. However, while there is evidence for GIPR engagement by tirzepatide in *ex vivo* human islets (El *et al*. 2023), the direct engagement of GIPR by tirzepatide *in vivo* in humans is still debated (Drucker & Holst 2023). Like the GLP-1R, the GIPR is Gαs-coupled, with cAMP production and insulin secretion promoted downstream of its activation, which also leads to glucagon secretion from alpha cells, adipose tissue lipid deposition, and a reduction in bone resorption (Mayendraraj *et al*. 2022). On the other hand, GCGR is found primarily in the liver and kidney; in hepatocytes, where it is also predominantly Gαs-coupled (McGlone *et al*. 2021), GCGR cAMP signalling leads to PKA activation and phosphorylation of cAMP response element binding protein (CREB), which increases transcription of glucagon-activated genes such as phosphoenolpyruvate carboxykinase 1 (PCK1) and glucose-6-phosphatase (Cajulao *et al*. 2022), resulting in increased hepatic glucose production via simulation of glycogenolysis and gluconeogenesis, as well as hepatic amino acid uptake and ureagenesis (Marroqui *et al*. 2014), while reducing hepatic fat accumulation by increasing fatty acid oxidation and decreasing *de novo* lipogenesis (Longuet *et al*. 2008). GLP-2R is expressed mainly in the gastrointestinal tract and regions of the central nervous system. GLP-2R signalling, following activation by GLP-2, is mainly via Gαs coupling causing an increase in cAMP accumulation leading to increased intracellular calcium and expression of cell survival genes triggering intestinotrophic effects (Drucker & Yusta 2014, Gadgaard *et al*. 2022, 2023). As a result, agonists of the GLP-2R such as teduglutide have been used for the treatment of short bowel syndrome and other intestinal conditions (Bahrami *et al*. 2010, Drucker & Yusta 2014, Gadgaard *et al*. 2023).

All four receptors, GLP-1R, GIPR, GCGR, and GLP-2R, belong to the class B1/secretin-like group of G protein-coupled receptors (GPCRs), integral membrane proteins that can detect a vast array of extracellular signals and transmit them into the cells via a range of G protein signalling pathways. GPCRs are divided into four major classes, rhodopsin-like (class A), secretin-like (class B), glutamate (class C), and frizzled (class F) (Latorraca *et al*. 2017, Yang *et al*. 2021).

**Figure 1:**
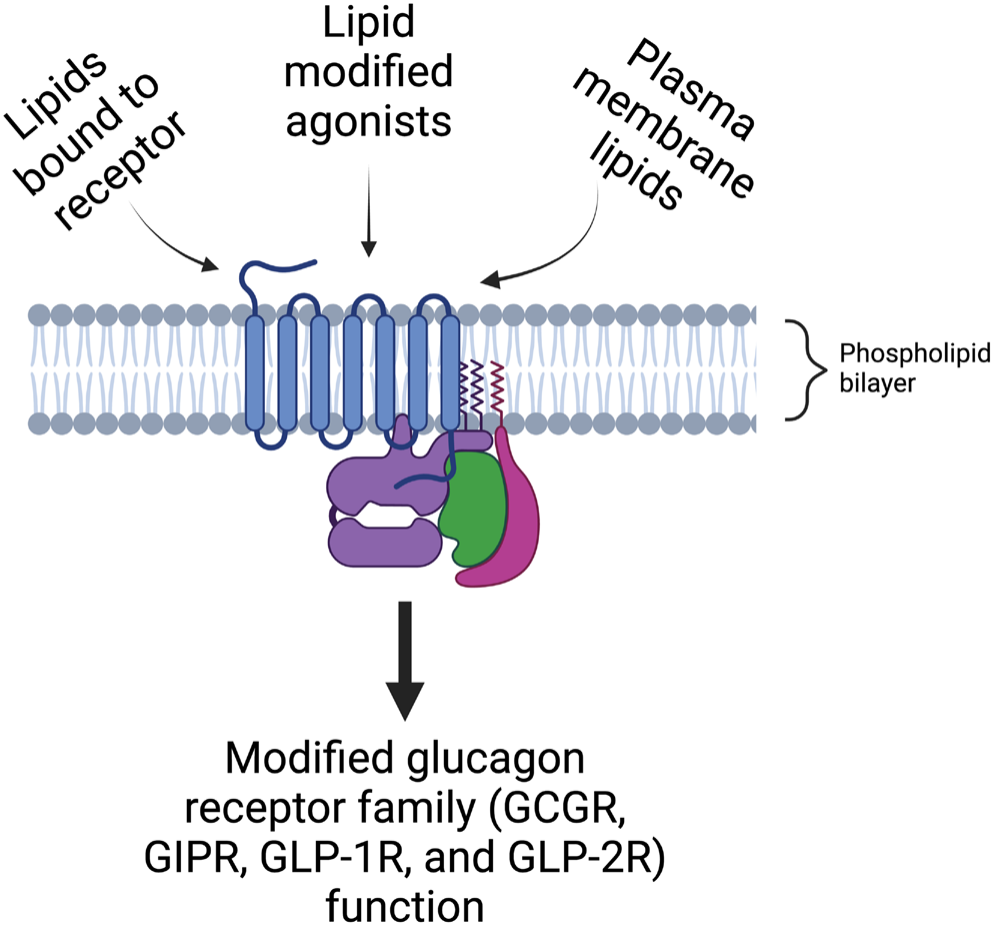
Schematic diagram summarising the potential effects of lipids on glucagon receptor family function.

The glucagon family of receptors has a typical class B1 GPCR structure, with seven transmembrane domains (TMD), three extracellular and intracellular loops, a large N-terminal extracellular domain (ECD), and an intracellular C-terminal tail. Being embedded in the plasma membrane, they interact intimately with lipids, and their function is greatly affected by changes in the lipid microenvironment. There is extensive evidence to support the effect that lipids have on GPCR function, with cholesterol specifically acting as an allosteric modulator (Oates & Watts 2011, Kumar & Chattopadhyay 2021, Yeliseev *et al*. 2021, Baccouch *et al*. 2022). However, much of the work done to investigate lipid regulation of GPCR functions has focused on other GPCR classes, as highlighted in Table 1, with our understanding of lipid modulation of class B1 GPCRs only beginning to be explored. This review will focus on highlighting the effects that lipids have on the function of the glucagon receptor family (Fig. 1), including their importance in the development of treatments for type 2 diabetes and obesity, as well as a description of current techniques to study lipid–receptor interactions, as well as areas that need to be further explored.

**Table 1:** Effects of lipids on the modulation of GPCR function.

Class	GPCR	Lipid	Evidence	References
A	β_2_-Adrenergic receptor (β_2_AR)	Cholesterol	Cholesterol interacts with receptor; stability; allosteric modulation; dimer formation	(Hanson *et al*. 2008, Manna *et al*. 2016, Cherezov *et al*. 2007)
		Phospholipids	Modulator of receptor structure and activation	(Dawaliby *et al*. 2016, Neale *et al*. 2015)
	A_2A_ adenosine receptor (A_2A_AR)	Cholesterol	Cholesterol interacts with receptor; cAMP modulation	(Liu *et al*. 2012, McGraw *et al*. 2022)
	μ-opioid receptor (OPRM1)	Cholesterol, palmitoylation	Receptor homodimerisation; receptor-Gαi_2_ coupling modulation	(Zheng *et al*. 2012)
	Cannabinoid receptor 1 (CB_1_R)	Cholesterol	Cholesterol interacts with receptor; modulates agonist binding; modulates G protein signalling via the adenylate cyclase and MAPK pathways	(Hua *et al*. 2017, Bari *et al*. 2005, Oddi *et al*. 2011)
	Cannabinoid receptor 2 (CB_2_R)	Cholesterol	Modulates basal activation of receptor	(Yeliseev *et al*. 2021)
B	Glucagon-like peptide-1 receptor (GLP-1R)	Cholesterol, palmitoylation	Receptor internalisation, receptor clustering, lipid raft recruitment, modulates cAMP production	(Buenaventura *et al*. 2019)
	Glucagon receptor (GCGR)	Cholesterol, PI(4,5)P_2_	Cholesterol predicted to interact with receptor, modulates cAMP production; PI(4,5)P_2_ potentially stabilises GCGR inactivate state	(McGlone *et al*. 2022)
	Glucose-dependent insulinotropic polypeptide receptor (GIPR)	Cholesterol, palmitoylation	Lipid raft recruitment	(Buenaventura *et al*. 2019)
	Glucagon-like peptide-2 receptor	Cholesterol	Receptor internalisation	(Estall *et al*. 2004)
C	Metabotropic glutamate receptor (mGlu1R)	Cholesterol	Cholesterol interacts with receptor; dimer formation; lipid raft recruitment	(Wu *et al*. 2014, Eroglu *et al*. 2003)
F	Smoothened receptor (SMO)	Cholesterol	Activation via its extracellular cysteine-rich domain	(Siebold & Rohatgi 2023, Xiao *et al*. 2017, Luchetti *et al*. 2016)

## Direct lipid regulation of the glucagon receptor family

**Figure 2:**
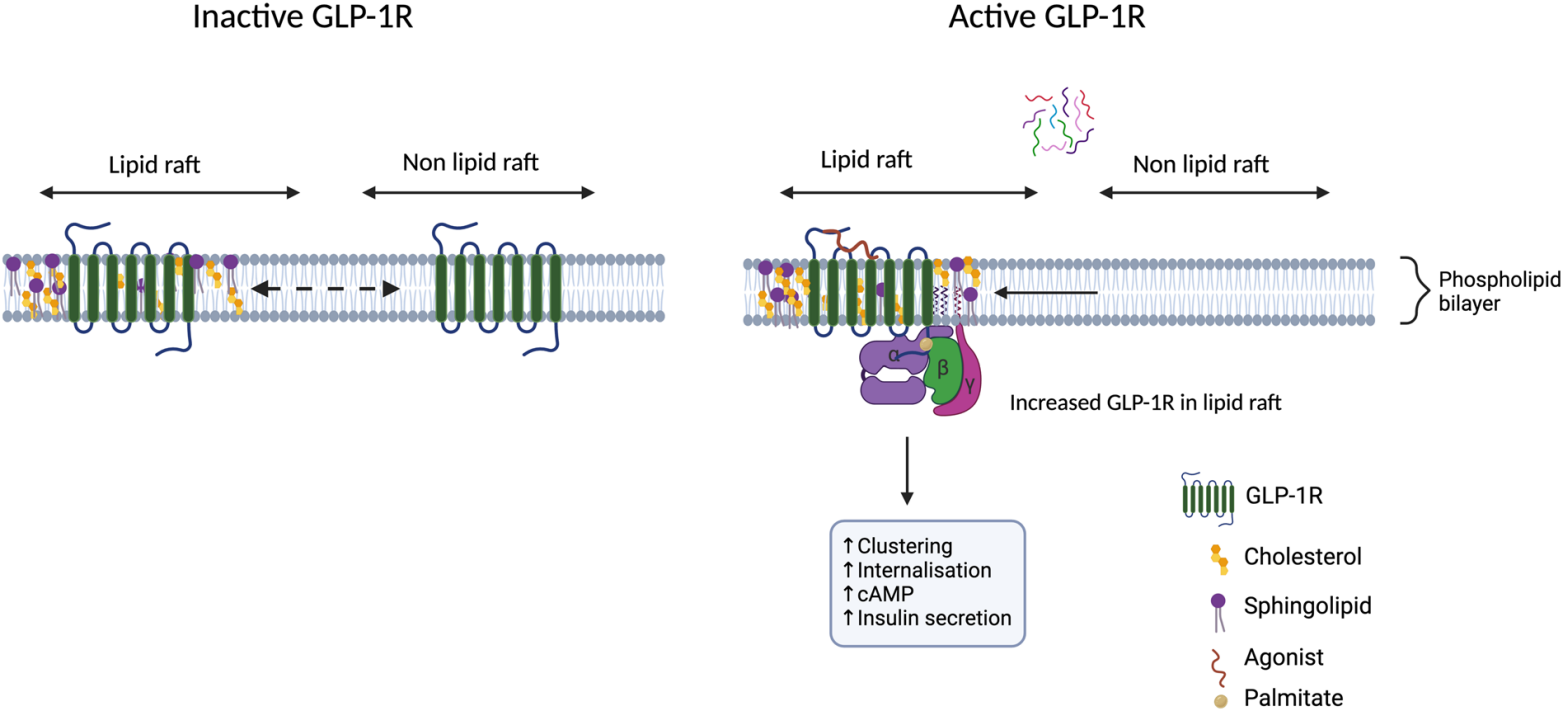
Schematic of GLP-1R recruitment to lipid rafts and relevance for the control of its signalling.

The plasma membrane is composed of a complex matrix of lipids, including glycerophospholipids (65%), sphingolipids (10%), and cholesterol (25%), and embedded proteins such as glycoproteins, ion channels, and other integral proteins like GPCRs (van Meer & de Kroon 2011, Koldsø *et al*. 2014). The structure and fluidity of the plasma membrane allows for the maintenance of the cellular architecture and is key in the regulation of membrane trafficking and organisation of its factors in different subdomains such as cholesterol-rich lipid rafts. Lipid rafts (also known as lipid nanodomains) are highly organised but dynamic, detergent-resistant membrane subdomains formed by the selective interaction of certain sphingolipids and cholesterol with specific membrane proteins required for signalling. These subdomains are important for the regulation of receptors like GPCRs, playing key roles in their sorting, trafficking, and signalling (Klose *et al*. 2013, Koldsø *et al*. 2014, Buenaventura *et al*. 2019, Kwiatkowska *et al*. 2020), including for the glucagon receptor family. Studies in pancreatic beta cells have shown that the GLP-1R relocates to detergent-resistant membrane fractions after stimulation with GLP-1 and the peptide agonist exendin-4 (Fig. 2). This was further confirmed via time-resolved (TR)-Förster resonance energy transfer (FRET), using Lumi4-TB labelled SNAP-tagged GLP-1R as the donor and the solvatochromic probe NR12S, which emits a blue-shifted wavelength when in a liquid-ordered membrane environment, as the acceptor in response to exendin-4 stimulation. This segregation was accompanied with increased GLP-1R clustering, measured using electron microscopy and total internal reflection fluorescence photoactivatable localisation microscopy (PALM) experiments. Disruption of the plasma membrane architecture using methyl-β-cyclodextrin (MβCD), which sequesters cholesterol, caused a dose-dependent reduction in receptor binding affinity to a fluorescent exendin-4 derivative due to faster agonist dissociation, leading to reduced cAMP signalling and inhibition of GLP-1R internalisation. Of note, given that GLP-1R signals mainly through Gαs, this G protein subunit was shown to preferentially partition into detergent-resistant membrane fractions, highlighting the importance of lipid domain organisation for the regulation of GLP-1R signalling (Buenaventura *et al*. 2019).

The underlying mechanism of GLP-1R recruitment to lipid nanodomains has not been fully elucidated, but it was shown to involve specific receptor post-translational modifications (PTMs), which are covalent additions of a modifying group with important effects for receptor structure, localisation, and function. Amongst the different forms of GPCR PTMs, palmitoylation, also known as S-acylation, is a lipid PTM which involves the addition of a palmitate moiety by covalent linkage to cysteine residues, important for the localisation of transmembrane proteins to lipid rafts. GPCRs are usually palmitoylated in C-terminal tail cysteine residues (Levental *et al*. 2010, Baccouch *et al*. 2022). An increase in GLP-1R palmitoylation was detected after stimulation with the endogenous agonist GLP-1 as well as with exendin-4. GLP-1R contains three cysteine C-terminal residues (C438, C458, and C462), which could potentially be involved in GLP-1R palmitoylation, with the cysteine in position 438 previously shown to undergo palmitoylation (Vázquez *et al*. 2005). Consistently, a C438A point mutation in GLP-1R disrupted its palmitoylation without affecting receptor surface expression levels or binding affinity to fluorescently labelled exendin-4 when compared with the wild-type receptor. The palmitoylation mutant was also associated with delayed exendin-4-mediated receptor clustering, reduced recruitment to detergent-resistant membrane fractions, reduced internalisation, and reduced cAMP response and insulin secretion (Vázquez *et al*. 2005, Buenaventura *et al*. 2019). These findings highlight the importance of lipid PTMs on the control of GLP-1R association with specific lipid nanodomains to regulate its function, including glucose homeostasis.

Like GLP-1R, GIPR has also been found to localise to flotillin-positive, detergent-resistant membrane fractions in pancreatic beta cells, indicating the likely presence of the receptor in lipid rafts. However, unlike the GLP-1R, the GIPR seems to be constitutively present in rafts, and increased agonist-induced translocation to lipid nanodomains was not detected. Furthermore, GIPR was found to be constitutively palmitoylated in contrast to GLP-1R, which showed increased palmitoylation upon agonist stimulation. These findings appear to be aligned with previous results indicating that GIPR is more active at basal states compared to GLP-1R (Al-Sabah *et al*. 2014). There is currently limited information about the interaction of GIPR with plasma membrane and other lipids with regard to its regulation. However, given that GIPR basal activity mirrors its membrane lipid nanodomain localisation and palmitoylation status, suggesting the likely relevant functional importance of these interactions, it will be important to further investigate the regulation of GIPR by cellular lipids in the future (Al-Sabah *et al*. 2014, Buenaventura *et al*. 2019, Manchanda *et al*. 2023).

With regard to the effects of lipids on the regulation of GCGR function, it is worth noting that resistance to glucagon has been reported in patients with metabolic-associated steatotic liver disease (MASLD) (Suppli *et al*. 2020), although the mechanism underlying this effect is not completely understood. The lipid composition of the cellular membranes of hepatocytes is altered in patients with hepatic steatosis (Gorden *et al*. 2011); indeed, the degree of hepatic cholesterol accumulation correlates with the severity of MASLD (Puri *et al*. 2007). One possible explanation is that certain membrane lipids might act as allosteric modulators of the GCGR, modifying receptor outputs following their accumulation. Downstream signalling of this receptor is known to be negatively allosterically modulated by the accessory protein receptor-activity modifying protein 2 (RAMP2) (McGlone *et al*. 2021, Krishna Kumar *et al*. 2023), but evidence of a direct allosteric effect of specific lipids on the GCGR is yet to be demonstrated.

A recent combined mass spectrometry and molecular dynamics (MD) simulation study has revealed that GCGR binds with high affinity to lipids with a phosphatidylinositol 4,5-bisphosphate (PI(4,5)P_2_) headgroup (Kjolbye *et al*. 2022). The PI(4,5)P_2_ tail composition determines its binding affinity, which is increased for stearic and arachidonic fatty acid tails. Interestingly, the binding site of the high-affinity saturated 16:0/18:1 PI(4,5)P_2_, between TM6 and TM7 of GCGR, is very similar to that of the negative allosteric modulator NNC0640 (PDB 5XEZ) (Zhang *et al*. 2017). This could indicate that 16:0/18:1 PI(4,5)P_2_ can stabilise the inactive conformation of GCGR (Kjolbye *et al*. 2022). GCGR also binds to several other phospholipids in MD simulations, including phosphatidylglycerols (PGs; (Kjolbye *et al*. 2022)). As PGs are present in human and murine hepatocytes, and the relative distribution of PG species changes in disease states of steatosis and cirrhosis (Gorden *et al*. 2011), this interaction, if validated, could be important to determine the functional state of the GCGR under these conditions. It remains to be determined if such interactions with PI(4,5)P_2_ are also relevant for other members of the glucagon receptor family.

Cholesterol affects deformability and curvature of the lipid membrane, thereby modulating the agonist binding and activity of GPCRs (Harayama & Riezman 2018), and can also directly allosterically modulate certain GPCRs (van Aalst & Wylie 2021). Manipulating cholesterol levels in hepatocytes affects GCGR sensitivity – increasing cholesterol decreases cAMP production, and vice versa (McGlone *et al*. 2022). This relationship holds true in mouse models where liver cholesterol is manipulated using cholesterol and statin diets (McGlone *et al*. 2022). Whether this is due to a direct negative allosteric effect of cholesterol binding on GCGR signalling is yet to be established. Although the GCGR contains cholesterol-recognition amino-acid consensus (CRAC) and inverted CRAC (or CARC) motifs, which have been proposed to take part in cholesterol interactions, the presence of such motifs is neither sensitive nor specific to determine receptor cholesterol-binding sites (Sejdiu & Tieleman 2020). Recent MD simulations using the protein-lipid analysis toolkit, PyLipID, have however predicted several likely GCGR cholesterol-binding sites (McGlone *et al*. 2022). Two of these are related to the G protein binding site, which involves TM6. Experimental cholesterol loading reduced glucagon-stimulated recruitment of mini-Gs, a conformational biosensor for Gαs favouring active GPCR conformations, indicating that cholesterol might act as a negative allosteric modulator for the GCGR (McGlone *et al*. 2022). Further investigations using mutagenesis techniques are warranted to explore this possibility. Additionally, similar MD simulations could be applied to the rest of the glucagon receptor family to identify and screen potential cholesterol binding sites for effects in receptor function and suitability for allosteric modulation.

Finally, Estall *et al*. showed that, similarly to GLP-1R and GIPR, sequestering of cholesterol using MβCD or filipin significantly decreased GLP-2R internalisation after stimulation with GLP-2. However, unlike the GLP-1R, the disruption in internalisation due to cholesterol sequestering did not affect GLP-2R-dependent cAMP accumulation. GLP-2R was also shown to localise to plasma membrane lipid raft domains in vehicle conditions, a localisation which was increased after stimulation with GLP-2 (Estall *et al*. 2004). This further highlights the importance of lipids on the regulation of the function of all members of the glucagon receptor family. However, how these lipids, especially cholesterol, regulate GLP-2R function still needs to be thoroughly investigated.

## Indirect lipid regulation of the glucagon family of receptors

Aside from the effects that specific membrane lipids have on the glucagon receptor family, lipid modifications of receptor *agonists* themselves, and the resulting effect that this might have on the nature of the agonist-membrane interactions, can also play an important role in modulating receptor binding kinetics and, in turn, receptor function. Incorporation of fatty acid-like moieties is a highly successful strategy that prolongs the circulatory half-lives of therapeutic peptides by allowing reversible binding to albumin, thereby preventing renal filtration via size exclusion (Knudsen & Lau 2019). Injudicious placement of the lipid modification directly affects (usually detrimentally) productive interactions between the ligand and the receptor, and there is also emerging evidence that the lipid can engage in primary interactions with the plasma membrane which, in turn, influences subsequent binding events with target receptors in the immediate vicinity. Table 2 lists the different lipid modified peptide agonist targeting the glucagon family of receptors that are currently available or being investigated.

**Table 2:** Lipid modified peptide agonists targeting the glucagon family of receptors.

Agonist	Receptor Target	Lipid Modification	References
Liraglutide	GLP-1R	C16 fatty diacid chain	(Zhao *et al*. 2022)
Semaglutide	GLP-1R	C18 diacid linked with a γGlu-2xOEG linker	(Lau *et al*. 2015)
Peptide 19	GLP-1R and GIPR	C18 Acetyl moiety	(Zhao *et al*. 2022)
Tirzepatide	GLP-1R and GIPR	C20 fatty diacid moiety linked with γ-Glu-2xAdo	(Coskun *et al*. 2018, Chavda *et al*. 2022)
MEDI0382	GLP-1R and GCGR	C16 palmitic acid with a γ-carboxylate spacer	(Li *et al*. 2023)
SAR425899	GLP-1R and GCGR	C16 palmitic acid with a γ-carboxylate spacer	(Li *et al*. 2023)

Lixisenatide, an analogue of exendin-4 with six lysine residues attached to its C-terminal tail, has a faster association rate constant compared to other receptor ligands, including exendin-4 (Zhao *et al*. 2022). This was proposed to be linked to the basic nature of the poly-lysine residues, which tend to bind to the plasma membrane, alluding to the role of lipids within the plasma membrane to enable this effect. Other agonists which display long residence times and varied GLP-1R outputs due to the effect of their lipid modifications include liraglutide, an analogue of GLP-1 with a C16 fatty diacid chain, and peptide-19, a dual GLP-1R and GIPR agonist with high potency at both receptors (Johnson *et al*. 2021), with these lipid modifications contributing to and modifying their binding properties compared to GLP-1 (Zhao *et al*. 2022). As long residence times are often due to slow agonist dissociation rates, we can speculate that the interaction between the lipid moiety and the cell membrane might preserve a heightened local concentration of agonist close to the membrane-embedded receptors which might contribute to explain these effects.

Other lipid-modified GLP-1R agonists with varying effects on receptor binding kinetics and function include semaglutide, a GLP-1 analogue similar to liraglutide but with two amino acid substitutions (Aib8, Arg34), which is derivatised at lysine 26 with a C18 diacid with a γGlu-2xOEG linker, as well as the dual GLP-1R/GIPR agonist LY3298176, also known as tirzepatide, which also has a fatty acid modification and varied effects on GLP-1R function (Coskun *et al*. 2018, Zhao *et al*. 2022). The lipid modifications in semaglutide have been proposed to cause increased agonist-membrane interactions, leading to increased receptor signalling (Lau *et al*. 2015, Zhao *et al*. 2022). These agonists are both clinically relevant and currently FDA-approved for type 2 diabetes, with semaglutide further approved for weight loss therapy (Lau *et al*. 2015, Coskun *et al*. 2018, Knudsen & Lau 2019, Frías *et al*. 2021).

Further investigations of acylated analogues of exendin-4, as well as with differentially biased GLP-1R agonists exendin-F1 and exendin-D3, have highlighted the importance of agonist lipid modifications on GLP-1R activity. Acylation of these analogues included the addition of C16 diacid at the C-terminal end through a GK linker. The exendin-4-C16 analogue caused an increase in plasma membrane interactions, accompanied with reduced GLP-1R internalisation, bias towards G protein recruitment and increased insulin secretion. The exendin-F1-C16 analogue had high affinity for the receptor compared to its non-acylated counterpart, which was accompanied by reduced internalisation and cAMP potency compared with exendin-D3-C16. Overall, lipid modifications of GLP-1R agonists appear to contribute to different agonist–receptor–plasma membrane interactions and favour specific receptor conformations, thereby contributing to modified or biased signalling (Lucey *et al*. 2020, 2021).

There are other lipid-modified agonists which target the GLP-1R, however these also target the GCGR, making them GLP-1R/GCGR dual agonists. Li *et al*. recently highlighted the importance of lipid modifications on the function of two dual peptide agonists, MEDI0382 and SAR425899, on GLP-1R and GCGR function. MEDI0382 and SAR425899 are both palmitoylated with a 16-carbon palmitic acid with a γ-carboxylate spacer at lysine 10 and 14, respectively. Lipidation of these dual agonists stabilised the binding of the peptide through increased interactions with the receptors and the plasma membrane. Non-lipidated versions of each peptide caused a significant decrease in cAMP accumulation for GLP-1R and essentially no cAMP accumulation for GCGR, highlighting the importance of these lipid modifications on altering both GLP-1R and GCGR signalling (Li *et al*. 2023). There is comparatively less information on the effects on lipid modifications of GCGR agonists on GCGR function alone.

A study investigating the effects of acylation of GLP-2 also highlights the importance of agonist lipid modification on GLP-2R function. Trier *et al*. showed that increased acyl chain length on GLP-2 caused enhanced partitioning into POPC simplified lipid membranes. This increase in membrane interaction was also established within a complex cell plasma membrane environment. The difference in acyl chain lengths also caused changes in translocation across intestinal cells when compared to non-lipid modified GLP-2 (Trier *et al*. 2014). Gadgaard *et al*. on the other hand, investigated the best position/residue for lipidation and maintained the same length of the acyl chain. GLP-2R internalisation, cAMP accumulation, and β-arrestin 1 and 2 recruitment were significantly reduced when agonists were lipidated at the N or C terminus, while changes in the middle of the agonist did not significantly affect receptor function (Gadgaard *et al*. 2023). These results highlight the importance of not only the type of lipid modification but also the location of this change on downstream receptor function.

It is worth noting, however, that certain lipid modifications of glucagon receptor family agonists could potentially cause biased receptor reactions or agonist-membrane interaction effects that might not always be as favourable as for some of the modifications mentioned previously.

There is presently far less information available about how lipid modification of GIPR agonists alters GIPR function or interactions with membrane lipids, probably as a result of the relatively recent interest in GIPR as a therapeutic target in type 2 diabetes and obesity compared to the GLP-1R. Currently, the focus has been on conferring an increased half-life to agonists in order to assess chronic GIPR agonism (Lafferty *et al*. 2023). The introduction of 2-aminoisobutyric acid and fatty acylation to GIPR agonists confers DPP4 resistance and enhanced albumin binding (Mroz *et al*. 2019), however whether these lipid modifications lead to altered GIPR signalling is currently unknown.

Another indirect lipid-induced regulation of GLP-1R involves the use of endocannabinoid-like lipids (Cheng *et al*. 2015). These lipids, which include oleoylethanolamide (OEA) and 2-oleoylglycerol (2-OG), are known to regulate food intake, potentially via increased GLP-1 release, and are structurally similar to endocannabinoids but with saturated or monounsaturated instead of polyunsaturated fatty acids, and without activating cannabinoid receptors. These are circulating lipids present in varying amounts in different tissues, with their levels being modified on demand via dietary changes in their membrane phospholipid precursors (Cheng *et al*. 2015, Rahman *et al*. 2021). Cheng *et al*. showed that both OEA and 2-OG can bind to GLP-1 without disrupting GLP-1 binding to GLP-1R, in a dose dependent manner, increasing the potency of GLP-1R-mediated cAMP production in the rat insulinoma RINm5F cell line. cAMP responses were not observed with the fatty acids alone or using the GLP-1R antagonist exendin-9, indicating their specificity in targeting the GLP-1R. As these lipids are differentially expressed within different tissues, they have the potential for spatial regulation of GLP-1R responses. This study also highlights their relevance as potential type 2 diabetes therapies (Cheng *et al*. 2015).

In normal conditions, GLP-1R activation by GLP-1 enhances glucose stimulated insulin secretion (GSIS) by potentiating the closing of ATP-sensitive potassium channels, leading to membrane depolarisation, which in turn causes an increase in intracellular calcium through the opening of voltage-gated Ca^2+^ channels and mobilisation of intracellular Ca^2+^ stores required for the exocytosis of insulin secretory vesicles (Manchanda *et al*. 2021). An earlier study highlighted the localisation of certain channels to plasma membrane lipid rafts as indirectly affecting GLP-1R function. Specifically, GLP-1 potentiation of GSIS was affected by the raft localisation of the L-type Ca^2+^ channels Cav1.2 and Cav1.3, with its disruption resulting in a significant reduction in GLP-1-mediated cAMP accumulation and GSIS potentiation (Jacobo *et al*. 2009). This observation highlights the importance that these lipid nanodomains have as organisers of membrane-protein microarchitecture for effective signal transmission.

Other than indirect effects of lipids via lipid modified agonists, changes in dietary lipids, specifically free fatty acids (short and/or long) and 2-monoacyl glycerol (2-MAG), can also modify GLP-1R function indirectly by regulating GLP-1 secretion. Free fatty acid-induced changes in cytosolic Ca^2+^ concentration have been shown to regulate GLP-1 secretion (Hirasawa *et al*. 2005, Abdalqadir & Adeli 2022). Activation of the GPCR GPR40 by long-chain fatty acids and GPR119 by 2-MAG have also been identified as involved in regulating GLP-1 secretion (Müller *et al*. 2019, Drucker & Holst 2023). This, in turn, indirectly regulates GLP-1R activation and function. GIP secretion has also been proposed to be regulated by the activation of long-chain fatty acid receptors GPR120 and GPR40, thereby also indirectly regulating GIPR function (Sankoda *et al*. 2019). Short chain fatty acids have also been documented to stimulate both GLP-1 and (Abdalqadir & Adeli 2022) and GLP-2 (Tappenden *et al*. 2003) secretion. Varying effects of dietary lipids on glucagon secretion and in turn GCGR function have also previously been outlined by Galsgaard *et al*. (Galsgaard *et al*. 2019).

## Techniques to study lipid–receptor interactions

Characterising lipid–protein interactions *in vitro* is challenging, due to their dynamic nature and the need to use membrane mimetic environments. Structure-based approaches are one avenue for exploring specific GPCR–lipid interactions, as lipids and lipid-like species can be observed in X-ray crystallography and cryo-EM maps of GPCRs. Indeed, the progress made in recent years in our understanding of GPCR–lipid interactions can be partially attributed to the rise in GPCR structures solved by cryo-EM, allowing for co-purification with complex lipid compositions. Lipid-bound structures are now available for several class A and class C GPCRs (Duncan *et al*. 2020), however despite cryo-EM structures being solved for each of the 15 class B1 GPCRs since 2017 (Wootten & Miller 2020), only two of these have resolved lipid species. Six cholesterol molecules were modelled in the cryo-EM structures of PTH1R, solved by cryo-EM in detergent micelle (Zhao *et al*. 2019). Four cholesterol molecules were modelled into the CRF1R and CRF2R maps (Ma *et al*. 2020), which were also solved in detergent micelles. The cholesterol binding sites were found to overlap across these structures, with the authors of the CRF1R/CRF2R paper highlighting two conserved and well-resolved sites in TM4 helix and the ECD as possibly functionally important.

MD simulations have emerged as a powerful tool in the study of membrane proteins, as they allow the investigation of specific protein–lipid interactions at atomic or near-atomic resolution in model membranes (Corradi *et al*. 2018). Advances in software and hardware now allow the study of receptors in membranes of increasing complexity, towards ever-more realistic membrane models with asymmetric leaflet distributions (Marrink *et al*. 2019, Souza *et al*. 2021). Different levels of resolution have been used to study GPCR–lipid interactions, with much of the work centred around atomistic (atMD) and coarse-grained (cgMD) level resolution, which currently allows simulation around the microsecond range for nanometre membrane patches (Duncan *et al*. 2020). Using the cgMD Martini force field allows for longer timescales and system sizes compared to atMD, making it possible to measure statistically meaningful lipid binding affinities (Marrink *et al*. 2023). A key consideration in using these methods for GPCR–lipid interactions is that the Martini model has fixed secondary structure, and typically conformational changes are limited, whereas atMD simulations are limited by the shorter attainable simulation times (Borges-Araújo *et al*. 2023). Employing a multiscale approach combining cgMD and atMD analyses allows a more complete study of the GPCR conformational landscape (Hedger *et al*. 2019, Ansell *et al*. 2020).

High-throughput MD approaches have provided valuable insights into lipid interactions across GPCR families, with the open database ProLint containing representative lipid analyses from 20+ GPCRs (Sejdiu & Tieleman 2020). Intriguingly, the lipid sites observed in the cryo-EM structures of PTH1R (Zhao *et al*. 2019) and CRF1R/CRF2R (Ma *et al*. 2020) were not observed to bind cholesterol in these simulations. This may be due to the use of micelles during structure determination, or because the simulations are of a single state and do not include the ECD. Further work to incorporate the ECD and missing loops will be valuable for future studies, particularly as previous work has proposed that the ECD of GCGR is regulated by GM3, an area which should be further explored (Ansell *et al*. 2020), including for other receptors of the glucagon family. The advent of AlphaFold2 also allows more exploration of GPCR conformational spaces with models available for over 420 GPCRs in both active and inactive states on GPCRdb as of 2023 (Pándy-Szekeres *et al*. 2023). This opens up the possibility of extending the high-throughput approaches to find patterns in lipid interactions, including those involving multiple lipid species, hence using more complete models (Xu *et al*. 2021).

**Figure 3:**
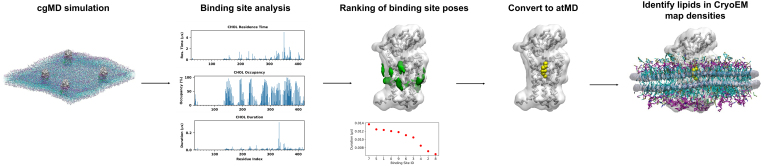
Example of a structure-based workflow to identify specific lipid binding sites of receptors.

A particularly powerful approach is to combine MD simulations with cryo-EM to correctly identify lipid-like cryo-EM map densities, by following a pipeline such as the one in Fig. 3, in which cgMD simulations are used to identify state-dependent lipid binding sites. This can be done using tools such as PyLipID, which finds bound poses for the identified lipid binding sites, and calculates residence times, average duration, and number of surrounding lipids of the individual protein residues (Song *et al*. 2022). The final ranked binding sites can be subjected to atMD simulations to compare with densities obtained from cryo-EM. This has been exemplified by the LipIDens pipeline, which allowed an informed assignment of the correct lipid species into the map density, enabling the identification of cholesterol during refinement of the membrane protein hedgehog acyltransferase (HHAT) (Ansell *et al*. 2022).

Simulations of the receptor of interest can be carried out in model membranes of given complexity. Coarse-grained simulations may be used for a state-dependent analysis. Specific lipid interaction metrics can be quantified using tools such as PyLipID or Volmap (Cohen *et al*. 2006) to calculate average lipid occupancy around the protein. Lipid binding sites and poses may then be ranked according to these metrics. Top poses which overlap with cryo-EM map densities may be converted to atomistic resolution for further sampling of the ligand binding site. These simulations may then be used as the basis for assigning lipid densities in cryo-EM maps of the receptor of interest for further refinement, as exemplified by the LipIDens pipeline.

We anticipate that advances in cryo-EM and MD simulation approaches will continue to expand our understanding of the lipid regulation of GPCRs. This will be of particular value in the class B1 GPCR field, where a focus area should be to determine class B1 GPCR structures solved in lipid-containing environments such as nanodiscs, which provide more realistic membrane environments than detergent micelles, and allow further insights into possible lipid regulation of the ECDs (Denisov & Sligar 2016), a technique that has so far not been employed to resolve any structure for this GPCR class.

Other techniques having increasing impact on our understanding of lipid regulation of membrane proteins are NMR and mass spectrometry (MS), including native MS (nMS), HDX-MS, and lipidomics approaches. MD simulations have proven to be a highly complementary technique when used in concert with nMS to identify lipid species and binding sites. As discussed earlier, in recent work on the GCGR, cgMD simulations using different membrane compositions were used to screen for lipids with high binding affinity for the receptor. PI(4,5)P_2_ was identified as a strong binder, and nMS was then used to confirm this (Kjølbye *et al*. 2022). nMS and MD together were used to investigate the effect of tail length and saturation on binding. Recent developments in the nMS field have shown that membrane proteins can be studied directly from the membrane with no need for detergent manipulation, paving the way towards *in situ* structural biology (Chorev *et al*. 2018). We note however that cholesterol has not been readily observed in nMS of membrane proteins (Kostelic *et al*. 2019): in this instance, alternative NMR approaches might be useful as they have shown promise in understanding cholesterol binding, with solid-state NMR recently used to characterise binding of cholesterol to the influenza M2 channel (Elkins *et al*. 2017).

Click chemistry is another method of directly studying lipid–protein interactions. There are different forms of click chemistry, with one of the most common being the copper(I)-catalysed alkyne–azide cycloaddition method. For the case of lipid–protein interactions, the use of a ‘photoactivated’ click cholesterol has been established; here the structure of cholesterol was modified to include a diazirine crosslinker activated by ultraviolet light irradiation, which then binds to the surrounding molecules, including proteins. The photoactivated cholesterol also includes an alkyne group which allows for its tagging using a fluorescent azide group via copper-catalysed alkyne–azide cycloaddition (Hulce *et al*. 2013). This technique can be combined with pulldown of a protein of interest (including tagged receptors) using affinity beads followed by SDS PAGE and western blotting or MS to determine the level of interaction with the labelled cholesterol under different conditions and agonist stimulations. A positive of this method, when optimised, is that it is less disruptive of the ‘natural’ order of the plasma membrane as the alkyne or azide groups are small and the interactions are established in its natural environment (Liang & Astruc 2011, Hulce *et al*. 2013, Hofmann *et al*. 2014, Chauhan *et al*. 2020). A current limitation of this type of click chemistry is that this does not allow for the live imaging of lipid–protein interactions as cells need to be lysed prior to analysis.

Another means of investigating lipid–receptor interactions is the use of fluorescently tagged lipids or lipid-binding proteins, allowing cross-correlation or co-localisation analysis of fluorescently labelled receptors and lipids. A recent development is the use of a bioorthogonal-based cholesterol probe combined with stimulated emission depletion (STED) super-resolution microscopy in living cells. Here the cholesterol is modified to introduce an azide group in position 24. Copper-free click chemistry is then used to fluorescently label the cholesterol using dibenzocyclooctyne (DBCO) which contains a linker between the dye and the reacting group (Lorizate *et al*. 2021). Another fluorescence-based method is the use of purified recombinant probes based on toxins including the domain D4 of Perfringolysin O (D4H*), Ostreolysin A (OlyA), or Anthrolysin O (ALO) fused to a fluorescent protein, which bind to cholesterol-rich membrane regions and serve as cholesterol biosensors for lipid–protein interactions (Skočaj *et al*. 2014, Lim *et al*. 2019, Johnson & Radhakrishnan 2021). However, any tags used in this type of experiments should be validated to have minimal effects on the functionality of the lipids and/or the receptor, and ideally experiments should be carried out in functionally relevant cells. As previously mentioned, the use of solvatochromic and/or environmentally sensitive fluorophores like NR12S or laurdan, which can change emission depending on their location in lipid ordered vsdisordered membrane environments, can be combined with fluorescently tagged receptors to resolve the cross-correlation behaviour of the receptor with the dye to investigate lipid–protein interactions using high resolution microscopy techniques like STED (Saxena *et al*. 2015, Nicovich *et al*. 2018, Schneider *et al*. 2018).

## Physiological and pathophysiological implications of lipids on the function of the glucagon receptor family

The incretin effect is greatly affected in patients with type 2 diabetes, with the insulinotropic influence of the GIP hormone being diminished in pancreatic beta cells and the effect of GLP-1 reduced. As previously mentioned, agonists of the GLP-1R which avoid breakdown by DPP4 have been developed as means of treatment for type 2 diabetes and obesity to restore this effect (Nauck *et al*. 2004, Graaf *et al*. 2016, Chia & Egan 2020). The glucagon family of receptors has been targeted for the treatment of various diseases involving abnormal lipid profiles, lipid content, or lipid metabolism (Mulvihill 2018, Seghieri *et al*. 2018, Galsgaard *et al*. 2019, Ammann *et al*. 2023, Nauck & Müller 2023). However, the physiological or pathophysiological implications of lipids themselves on the function of this family of receptors in their respective tissues still needs to be investigated. Although mostly speculative, some indirect physiological and pathophysiological implications on these receptors are outlined below.

Dyslipidaemia, usually characterised by increased levels of plasma triacylglycerols and cholesteryl esters (Goldberg 2001, Rhee *et al*. 2011, Klop *et al*. 2013, Athyros *et al*. 2018, Eid *et al*. 2019), is a common risk factor in patients with type 2 diabetes and obesity. Rhee *et al*. investigated the difference in LC/MS-based lipid profiles of a mixed cohort consisting of control (no diabetes) and cases (developed type 2 diabetes) and highlighted different triacylglycerol profiles between the two groups. Patients with increased risk of type 2 diabetes had more triacylglycerols of lower carbon number and double bond content compared with controls. The nature of these triacylglycerols, i.e. saturated and monosaturated fatty acids, was shown to be different up to 12 years before overtly developing the disease (Rhee *et al*. 2011). This indicates the potential of changes in triacylglycerol composition to impact GLP-1R and GIPR activities, thereby increasing the onset of type 2 diabetes directly or indirectly within this patient group. How the specific triacylglycerols identified in this study could potentially modify GLP-1R and GIPR function needs further investigation.

Hyperglucagonaemia is characterised by excess plasma concentration of glucagon and has been observed in individuals with MASLD (Albrechtsen *et al*. 2018, Grandt *et al*. 2023). MASLD is characterised by abnormal lipid accumulation in hepatocytes due to a combination of factors including lipotoxicity, diet, sedentary lifestyle, and genetics, amongst others (Zarghamravanbakhsh *et al*. 2021). It is likely that patients with MASLD have resistance to the actions of glucagon, resulting in increased circulating amino acids, which stimulate the pancreas to secrete further glucagon (Suppli *et al*. 2016, Wewer Albrechtsen *et al*. 2018). Since one of the actions of glucagon is to decrease liver fat accumulation, hepatic resistance to glucagon could contribute to the pathophysiology of MASLD. Alternatively, or additionally, lipid accumulation in hepatocytes (MASLD) could reduce their ability to respond to glucagon. Further investigation is required to properly understand the potential effects of increased liver fat content on hepatic GCGR function. A similar pathophysiology has also been identified involving increased fat accumulation in the pancreas, known as non-alcoholic fatty pancreas disease (NAFPD). NAFPD has received a lot of interest recently as it is associated with loss of beta cell function (Silva *et al*. 2021, Zhang *et al*. 2021). As the pancreas is one of the main locations for GLP-1R and GIPR function, we can speculate that this could have detrimental effects on the function of these receptors, with the potential effects that these lipid changes might have on receptor outputs warranting further examination.

Certain lipids and lipid derivatives have previously been implicated in disrupting physiological responses of incretin receptors. Specifically, increased plasma non-esterified fatty acids (NEFA) have been proposed to cause impaired beta cell function in type 2 diabetes. Kang *et al*., investigating the role of NEFA on disrupting incretin responses, showed that exposure of pancreatic beta cells and mouse islets to palmitate caused a reduction in the expression of *Glp1r* mRNA and GLP-1R protein levels without affecting *Gipr* levels (Kang *et al*. 2013). They also showed that islets isolated from diabetic mice, when compared with those from control mice, had reduced *Glp1r* and *Gipr* expression, and that this expression was partially restored after treatment with the lipid lowering agent bezafibrate. Treatment of INS-1E cells or mouse islets with palmitate also caused a reduction in both GLP-1R and GIPR potentiation of GSIS. This pattern was different for cAMP production and CREB phosphorylation in rat INS-1E vsmouse MIN6 cells, with palmitate causing a decrease in the former for both GLP-1R and GIPR but only for GLP-1R in MIN6 cells. This highlights the importance of increased NEFA on impaired beta cell function, which might be species-specific (Kang *et al*. 2013). A human study carried out by Astiarraga *et al.* also showed that acute increases in plasma NEFA levels via lipid infusion of healthy non-diabetic volunteers caused a decrease in incretin-induced potentiation of insulin secretion (Astiarraga *et al*. 2018, Chueire & Muscelli 2021). Tanabe *et al.* also noted that increased triglyceride levels impaired liraglutide efficacy in type 2 diabetes patients, potentially through a reduction in *Glp1r* expression (Tanabe *et al*. 2016), suggesting that the effects can be translated to human physiology.

## Conclusion and prospective future work

Current literature highlights the importance of lipids on the function of GPCRs, with cholesterol playing a direct role as a potential allosteric modulator as well as an indirect effect related to the segregation of these receptors to cholesterol-rich nanodomains in the plasma membrane. While most of the work done regarding lipids and the glucagon receptor family has revolved around incretin receptor regulation of lipid metabolism (Yaribeygi *et al*. 2021), there is currently sparse information about the regulation of GLP-1R and GLP-2R by lipids, and little to no information about the regulation of GIPR, either directly or indirectly due to lipid modification of GIPR agonists. While an initial prediction of cholesterol binding sites has been performed for the GCGR, there is currently no corresponding information for the GLP-1R, GLP-2R, or GIPR. Given the importance of lipid interactions for the regulation of GPCR function (Baccouch *et al*. 2022), mapping cholesterol and/or other lipid binding sites is fundamental to increase our current understanding of the biology of this key family of metabolically relevant receptors. Identification of relevant and specific lipid interaction sites should be attempted using multidisciplinary structural and computational biology approaches and taking into consideration active vs inactive receptor states.

There is still a lot of research that needs to be done to fully understand the effect that lipids have on regulating the specific functions of the glucagon family of receptors besides identification of cholesterol, sphingolipid, and/or other lipid-binding sites. Important areas that need to be addressed include (i) extensive functional characterisation of lipid binding in physiologically relevant systems and determination of the effects of altering interacting lipid levels on receptor function in relevant tissues; (ii) harnessing the information generated on the effects of lipid–protein interactions as potential targets for drug development for the treatment of type 2 diabetes, obesity, and/or fatty liver disease; and (iii) human studies to investigate the effect of changes in lipid levels, either through diet or pharmacologically, on receptor function, and potential effects on patient responses to agonists targeting this family of receptors.

There might be the possibility of exploiting interactions between lipids and the glucagon family of receptors to develop small allosteric modulators that target and mimic the effect of identified lipid-binding sites, allowing for the pharmacological manipulation of receptors to either increase or reduce their lipid association to modulate receptor activity. In this context, genetic variants of these receptors (Michałowska *et al*. 2022, van der Velden *et al*. 2022, Gao *et al*. 2023, Lagou *et al*. 2023) are already known to trigger specific signalling responses; it will be interesting to determine the effects that these variants might have on receptor–lipid interactions, potentially allowing the development of personalised medicine approaches which take into consideration specific lipid associations when devising the most beneficial treatment regime. Another aspect to consider is the potential impact that lipid-modifying drugs might have on patients who are concomitantly prescribed an agonist that target the glucagon family of receptors.

This is a promising area of research not only for the development of novel treatments for metabolic diseases but also for the better fundamental understanding of lipid regulation of class B1 GPCR behaviours.

## Declaration of interest

We declare no conflict of interest that could be perceived as prejudicing the impartiality of the study reported.

## Funding

This work was supported by grants from the MRC (MR/X021467/1, MR/R010676/1, MR/M012646), Diabetes UKhttp://dx.doi.org/10.13039/501100000361 (19/0006094), an EFSD/Boehringer Ingelheimhttp://dx.doi.org/10.13039/100001003 European Research Programme, a Lilly Research Award Program (LRAP), a Commonwealth PhD Scholarship to AO, and funding from the NIHR and the Academy of Medical Scienceshttp://dx.doi.org/10.13039/501100000691. AT, SR, BJ, and ERM have recently received a Wellcome Trusthttp://dx.doi.org/10.13039/100010269 Discovery Award (301619/Z/23/Z) to resolve all actionable cholesterol binding sites in the GLP-1R and the GCGR.
